# Sequestration of dead-end undecaprenyl phosphate-linked oligosaccharide intermediate

**DOI:** 10.1099/mic.0.001530

**Published:** 2025-01-31

**Authors:** Yaoqin Hong, Jilong Qin, Matthew Thomas Doyle, Peter Richard Reeves

**Affiliations:** 1 Biomedical Sciences and Molecular Biology, College of Medicine and Dentistry, James Cook University, Douglas, Queensland, Australia; 2 School of Life and Environmental Sciences, The University of Sydney, Camperdown, New South Wales, Australia; 3 Centre for Immunology and Infection Control, Queensland University of Technology, Herston, Queensland, Australia; 4 Institute for Molecular Bioscience, University of Queensland, St Lucia, Queensland, Australia; 5 Centre for Drug Discovery Innovation, The University of Sydney, Darlington, New South Wales, Australia; 6 Sydney Infectious Diseases Institute, Faculty of Medicine and Health, The University of Sydney, Darlington, New South Wales, Australia; 7 School of Medical Sciences, Faculty of Medicine and Health, The University of Sydney, Darlington, New South Wales, Australia

**Keywords:** O-antigen, polysaccharide, substrate fidelity, substrate preference, undecaprenyl phosphate, Wzx flippase

## Abstract

Most Gram-negative bacteria synthesize a plethora of cell surface polysaccharides that play key roles in immune evasion, cell envelope structural integrity and host–pathogen interactions. In the predominant polysaccharide Wzx/Wzy-dependent pathway, synthesis is divided between the cytoplasmic and periplasmic faces of the membrane. Initially, an oligosaccharide composed of 3–8 sugars is synthesized on a membrane-embedded lipid carrier, undecaprenyl pyrophosphate, within the cytoplasmic face of the membrane. This lipid-linked oligosaccharide is then translocated to the periplasmic face by the Wzx flippase, where it is polymerized into a repeat-unit polysaccharide. Structural alterations to the O-antigen repeating oligosaccharide significantly reduce polysaccharide yield and lead to cell death or morphological abnormalities. These effects are attributed to the substrate recognition function of the Wzx flippase, which we postulated to act as a gatekeeper to ensure that only complete substrates are translocated to the periplasmic face. Here, we labelled *Salmonella enterica* serovar Typhimurium group B1 with [^14^C] d-galactose. Our results showed that strains unable to synthesize the full O-antigen repeat unit accumulate significantly higher levels of Und-P-linked material (~10-fold). Importantly, this sequestration is alleviated by membrane disruption which opens the lipid-linked oligosaccharide at the cytosolic face to periplasmic ligation to support accumulation occurs at the cytosolic face of the membrane.

## Introduction

The synthesis of glycoconjugates on lipid carriers is a conserved anabolic process across all domains of life. In bacteria (excluding mycobacteria), undecaprenyl phosphate (Und-P) is the predominant lipid carrier involved in glycan and proteoglycan biosynthesis. This common carrier plays a crucial role in various pathways including peptidoglycan, capsular polysaccharides, exopolysaccharides (e.g. colanic acid), *Enterobacteriaceae* common antigens (ECA) and O-antigen in *Enterobacteriaceae* species [1]. Many bacterial surface polysaccharides are synthesized by the Wzx/Wzy-dependent pathway. In this system, the biosynthetic process begins with the addition of a sugar-phosphate to Und-P by an initial transferase, forming an Und-PP-linked sugar. The oligosaccharide repeating unit is then assembled sequentially and translocated by the Wzx flippase to the periplasm and then either polymerized by the Wzy polymerase or directly incorporated into lipopolysaccharide by the WaaL ligase in a competitive manner [2,6]. Both polymerization and ligation subsequently release Und-PP, which is then dephosphorylated to give Und-P, and recycled back to the cytoplasmic face of the membrane [7,8].

Relative to the well-established Gram-positive model species, the availability of Und-P is limited in the Gram-negative bacteria [9]. Mutations in surface polysaccharide biosynthesis genes often lead to cell death or severe phenotypic consequences, including compromised cell envelope integrity [10,21]. One plausible explanation for these effects is that the sequestration of the Und-P pool impairs the ability to meet the demands of various biosynthetic pathways, particularly peptidoglycan biogenesis. Indeed, the overproduction of Und-P has been shown to alleviate cell envelope defects, and this has been instrumental in revealing how limited lipid carriers are accountable for the fitness costs associated with halted polysaccharide biosynthesis [20]. Thus, the traffic in the competing pathways in which Und-P is shared is anticipated to be well managed, and this element has captivated interest in unravelling the underlying interactions governing Und-P flow.

**Fig. 1.**
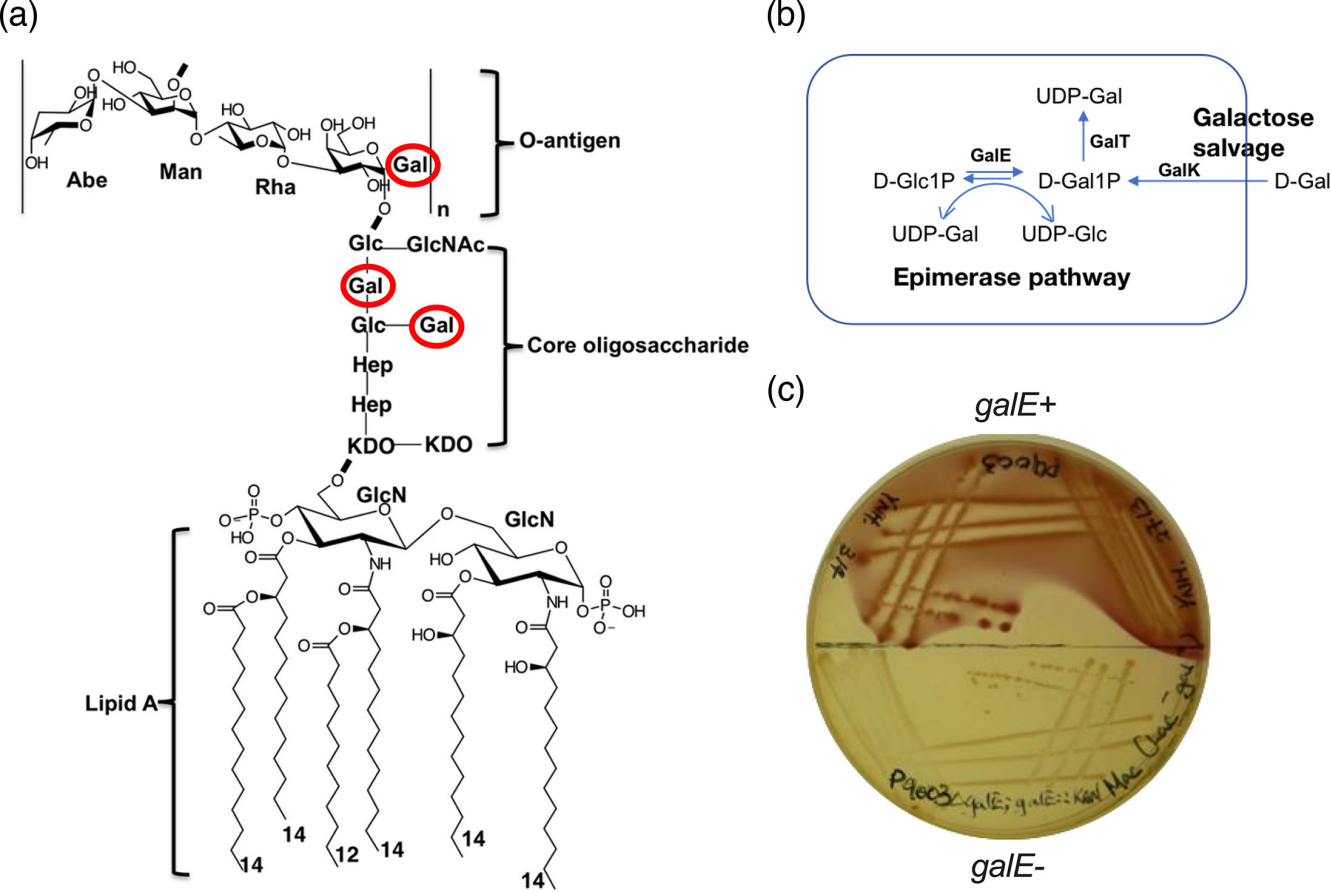
The structure of *S. enterica* group B1 LPS and controlled d-galactose metabolism. (a) Group B1 O:4 LPS structure. (**b)** d-Gal metabolism pathway. (**c) **Deactivation of *galE* to control d-Gal metabolism. d-Gal residue was circled in red to indicate biosynthetic steps blocked by the inaccessibility to the sugar precursor (pH indicator neutral red detects mixed acid fermentation and agar near bacterial growth would turn red if d-Gal is utilized). Abe, abequose; Man, mannose; Rha, rhamnose; Gal, galactose; GlcN, glucosamine.

Despite the importance of directly assessing Und-P levels, it has been challenging due to its relatively low abundance compared to other membrane lipids [22]. Indeed, the quantitative evidence for Und-P sequestration has primarily come from studies of *Escherichia coli* mutants with truncated ECA oligosaccharide repeat units [23]. Our previous research has demonstrated in *Salmonella enterica* groups B1 (used in this work), D2 and C2, which mutant strains producing truncated O-antigen repeat units exhibit minimal O-antigen production and have severe growth defects [13,14, 21]. To mitigate these effects, tactics involving controlled O-antigen production have been employed [13,14, 21]. Similarly, Nikaido *et al.* observed in 1969 that a group B1 mutant that produces an oligosaccharide without side-branch abequose and lacking biosynthetic control was unstable and would be replaced by revertants or suppressors after overnight growth [10]. For our *S. enterica* genetic studies [13,14, 21, 24], O-antigen defective strains were constructed in the *galE* null background. In these cases, O-antigen synthesis is controlled by the availability of exogenous d-Gal, the first sugar in all eight *S. enterica* Gal-initiated serogroups [25], and also an integral component of the outer core of lipopolysaccharide (Fig. 1a). The consequent rapid cell death upon O-antigen synthesis is attributed to substrate preference at the membrane translocation step, leading to the accumulation of Und-PP-linked material on the cytoplasmic face of the membrane and preventing effective recycling [14,21, 26]. Nonetheless, there should be no abnormal Und-P sequestration in these mutants prior to the addition of exogenous d-Gal. In this study, we employed radioactive [^14^C] d-Gal sugar labelling to investigate Und-P sequestration in O-antigen-defective mutants, which provided confirmatory support for the accumulation occurring on the cytosolic face of the cytoplasmic membrane.

## Methods

### Bacterial strains and growth conditions

The strains and plasmids used in this work are described in Table 1. Nutrient broth (sodium chloride 5 g l^−1^, yeast extract 5 g l^−1^ and bacteriological peptone g l^−1^) with sugar depletion carried out by the aerobic growth of MG1655 as previously described [13] was used for strain maintenance and assessment in this study. Media were supplemented with antibiotics as required at the following concentrations: ampicillin (25 µg ml^−1^), kanamycin (25 µg ml^−1^) and chloramphenicol (12.5 µg ml^−1^).

**Table 1.** Strain table

Strains	Parent strain	Descriptions	Source/reference
CL4419		*S. enterica* group B1 serovar Typhimurium strain LT2, *hsdL trp32 nml flaA66, rpsL’ xylT404 llvE452 metE551 metA22 hsdA*	(Ornellas EP, Stocker BA. Relation of lipopolysaccharide character to P1 sensitivity in *Salmonella typhimurium*. *Virology*. 1974;60(2):491–502.)
P9528	CL4419	CL4419 Δ*galE*::FRT	[13]
P9529	P9528	CL4419 Δ*galE*::FRT Δ*abe*::*kan*	[13]
P9545	P9529	CL4419 Δ*galE*::FRT Δ*abe*::*kan*/pPR618	[13]

### Assess UDP-galactose epimerase activity

Overnight grown single colonies were streaked onto modified McConkey agar containing 17 g l^−1^ tryptone, 3 g l^−1^ peptone, 10 g l^−1^ d-Gal, 1.5 g sodium deoxycholate, 5 g sodium chloride, 0.03 g neutral red (pH 7.2) and 13 g l^−1^ bacteriological agar. Growth using d-Gal as the carbon source reduces the pH of the medium due to mixed acid metabolism. In the presence of neutral red, the medium near the proximity of bacterial growth turns red indicating the ability to utilize d-Gal, while a negative colour change indicates no d-Gal utilization. Plates were incubated at 37 °C for 24 h before images were taken.

### [^14^C] d-Gal uptake assay

**Fig. 2.**
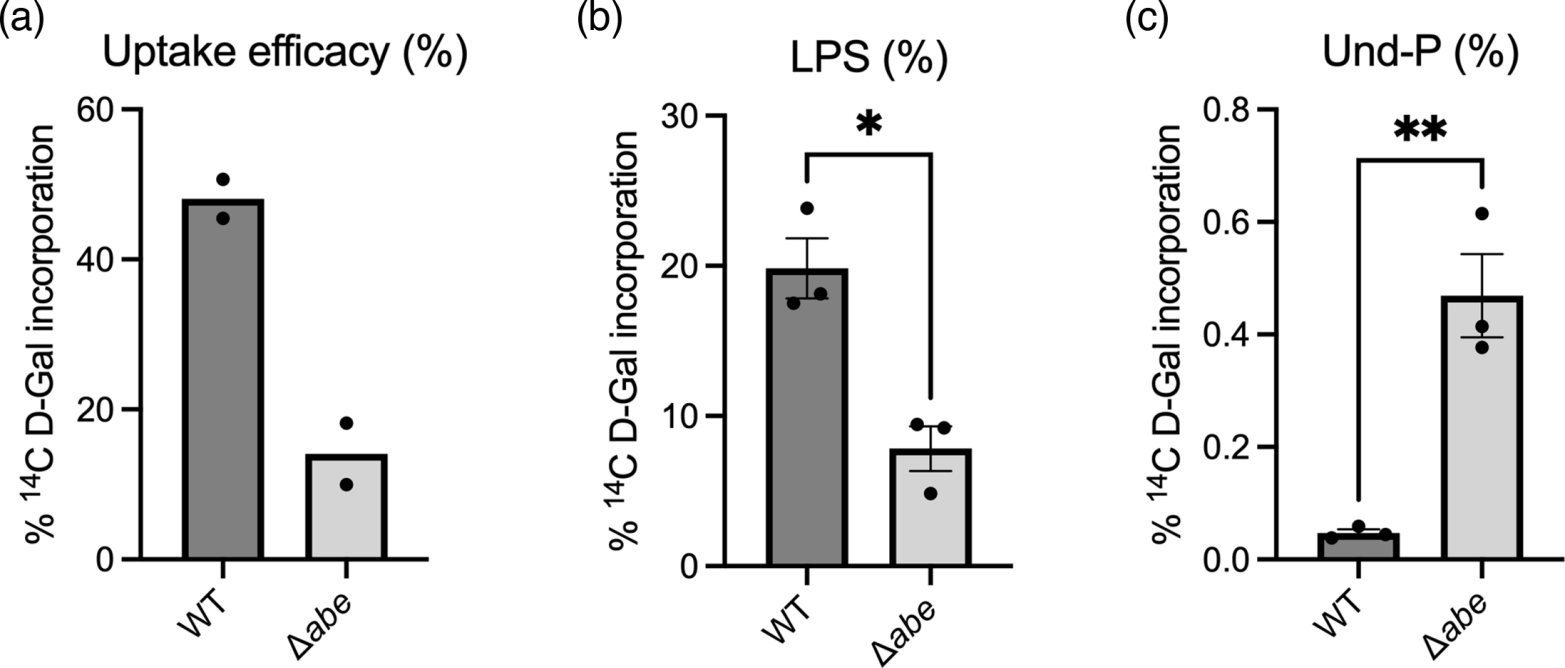
[^14^C] d-Gal uptake in *S. enterica* group B1 WT and ∆*abe*. (a) % [^14^C] d-Gal counts of the whole cell; (**b)** % [^14^C] d-Gal counts in purified LPS; (**c) **% [^14^C] d-Gal counts in n-butanol extracts. Data from independent repeats are shown, and the error bar uses sem.

Overnight broth cultures grown in depleted media were diluted 1:100 into 40 ml depleted media. The cultures were incubated at 37 °C until the mid-log phase. The cultures were then adjusted to equal OD_600_ (~0.5). [^14^C] d-Gal was mixed with 20% w/v d-Gal. The dilution gives a 35.37 Ci/mol specific activity that is adequately concentrated in signal intensity. A total of 250 nCi [^14^C] d-Gal that could provide sufficient substrate availability for the selected experiments described below was added per sample. Mid-log strain cultures with adjusted equivalent OD_600_ were fed with [^14^C] d-Gal for 15 min. The cell pellet was then collected and then rapidly chilled on ice. The pellet was then washed in 2 ml ice-cold 0.85% w/v saline. This sample was loaded into scintillation vials containing 5 ml ACSII scintillant and subjected to quantitative counting in Tri-Cab 2810 TR Liquid Scintillation Analyzer. The scintillation involved counting over 1-min intervals. The uptake range of the WT strain and the ∆*abe* is between 15 and 45% (Fig. 2a). As such, the majority of [^14^C] d-Gal was retained in the media, and the condition is suitable for subsequent assessments.

For assessing incorporation into the LPS and n-butanol extractable Und-PP-linked material, equivalent amounts of WT and ∆*abe* culture samples were prepared as above. The use of n-butanol in the extraction of Und-PP-linked material was first demonstrated by Robbins *et al.* [22]. Paper chromatography has validated the selective extraction of only phospholipid species and Und-PP-linked materials, with no detected peaks of LPS contamination or other materials, from WT *S. enterica* cells labelled with radioactive glucose, amino acids, glycerol and inorganic phosphate [22]. Given that [^14^C] d-Gal is only integrated into Und-PP-linked O-antigen intermediates and not phospholipids in n-butanol extract, the procedure served the purpose of selectively extracting Und-PP-linked O-antigen intermediates regardless of any, albeit unlikely, yet to be defined metabolic utilization of UDP-Gal in *S. enterica*. Each sample was divided into four equal 500 µl lots in 80 mM Tris-acetate (pH 8.0), 10 mM MgCl_2_ and 1 mM EDTA. Two lots were used for hot phenol water LPS extraction as previously described [13]. The other two lots were simultaneously added to equivalent volumes of 50 mM pH 8.0 KCl-buffered n-butanol to extract Und-PP-linked material as previously described [27,28]. The samples were then vortexed thoroughly to mix. Note that the samples were treated the same until enzymatic activities were deactivated with either phenol or n-butanol. The n-butanol fraction was back-extracted with 80 mM Tris-acetate (pH 8.0), 10 mM MgCl_2_ and 1 mM EDTA twice. One of the two lots used for LPS purification and n-butanol extractions was subjected to sonication. The samples were sonicated using a Branson Model 250/450 Stonefire (microtip) for 3 bursts of 8 s, with a 100% duty cycle and output control set to 3. Experiments were performed in three independent repeats.

## Results

### Controlling O-antigen synthesize via the availability of exogenous galactose

d-Gal is the first sugar of the O-antigen repeat unit in *S. enterica* Sv. Typhimurium (group B1) and an essential component of the outer core of lipopolysaccharide (Fig. 1a). *S. enterica* can acquire d-Gal through the biosynthetic epimerase (GalE) pathway or by salvage, in which exogenous d-Gal is utilized (Fig. 1b). We used a previously defined ∆*galE* null background in this work, in that the only source of UDP-Gal is through the GalK/GalT salvage pathway from the growth medium, thereby allowing the controlled initiation of O-antigen synthesis (Fig. 1b). In this genetic background, there should be no abnormal Und-P sequestration in the absence of salvageable d-Gal. We streaked CL4419 and its derived ∆*galE* mutant onto a d-Gal plate and confirmed that the strain is unable to utilize d-Gal as a carbon source (Fig. 1c). This observation validates the suitability to use [^14^C] d-Gal to label both the O-antigen and the core oligosaccharide. For simplicity, we will treat the ∆*galE* strain P9528 as our WT strain from here on.

### Sequestration of undecaprenyl phosphate in a group B1 O-antigen repeat unit mutant

The O-antigen repeat unit of group B1 *Salmonella* consists of a three-sugar mainchain (mannosyl–rhamnosyl–galactosyl) with an abequosyl side branch attached to the mannosyl residue (Fig. 1). The *abe* null mutant is unable to synthesize CDP-abequose, and such strains produce only the mainchain structure. Mutants carrying this defect were among the first strains to show lethality after the start of the O-antigen production [10]. Cells from WT and ∆*abe* strains, labelled with [^14^C] d-Gal under the described conditions, were used to quantify the uptake of [^14^C] Gal into LPS. We observed that the [^14^C] d-Gal content incorporated into the phenol-extracted LPS phase of the WT strain was ~3-fold higher than that of the ∆*abe* strain (ratio paired t-test, *P*=0.0319) (Fig. 2b). This finding aligns with our previous observations that the LPS produced by the ∆*abe* strain contains very little O-antigen and is mostly comprised of short-chain molecules [13,14]. We attributed this to most of this signal in the ∆*abe* mutant to be due to [^14^C] d-Gal incorporation into the LPS core, given that each complete core contains two d-Gal residues (see Fig. 1a).

Previously, we suggested that the truncated O-antigen intermediate produced by the ∆*abe* strain is sequestered at the intermembrane translocation step, and this interpretation had been instrumental in establishing Wzx flippase as the structural fidelity step in bacterial polysaccharide synthesis [13,14]. To confirm this, we extracted and quantified the Und-PP-linked O-antigen intermediate using n-butanol extraction. The results showed that the [^14^C] d-Gal counts in the n-butanol extract of the ∆*abe* strain were 10-fold higher than those in the WT extract (ratio paired t-test, *P*=0.0010) (Fig. 2c). This suggests that the WT counts represent the regular Und-P pool allocated to the intended O-antigen pathway, while the tenfold higher counts in the n-butanol extract of the ∆*abe* mutant indicate that a significant portion of lipid carriers are sequestered during the 15-min uptake period. We attributed the high n-butanol counts to the ineffectiveness of Wzx flippase at translocating the Und-PP-linked trisaccharide.

### Undecaprenyl phosphate sequestration occurred at the cytosolic face of the cytoplasmic membrane

**Fig. 3.**
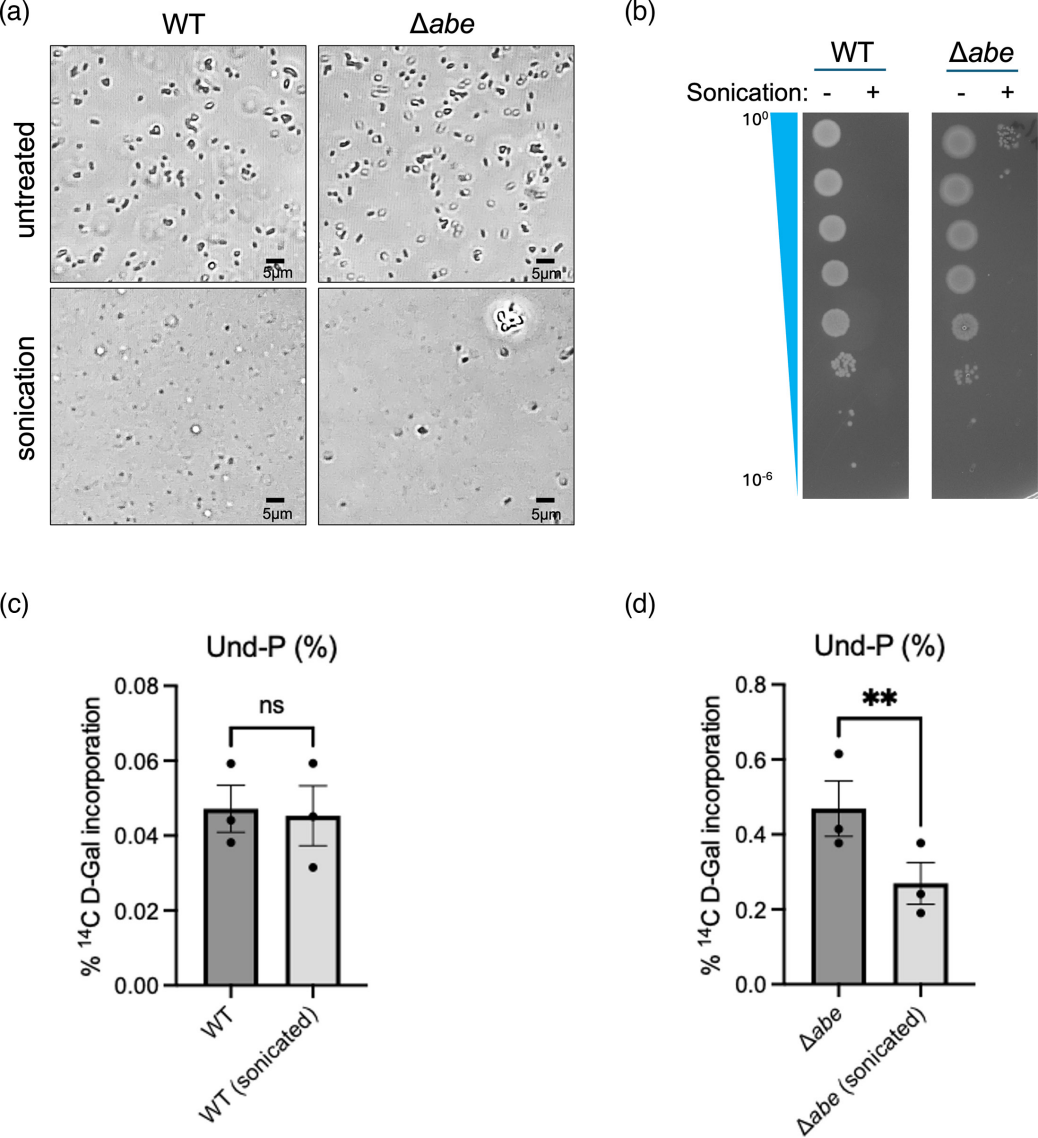
Reduction of [^14^C] d-Gal counts by membrane rearrangements in the n-butanol extractable pool from *S. enterica* group B1 ∆*abe* but not that from the WT pool. a Light microscopy image of WT and ∆*abe* samples after sonication.** (b) **Viability reduction by sonication. Both sonicated and untreated samples were adjusted to OD_600_ 1.0 and diluted in a tenfold series with fresh growth medium. Five microlitres from 10^0^ to 10^−6^ dilutions were spotted onto M9-glycerol medium supplemented with casamino acids, tryptophan and thiamine [14]. (**c) **Sonication does not affect the n-butanol extractable pool in the WT. (**d) **Sonication significantly reduced the n-butanol extractable pool in the ∆*abe* mutant. Note that the unsonicated n-butanol extractable pool counts in** (c, d) **were the same datasets as Fig. 2c. Growth and sonication treatment of cells in** (a, b) **were executed as described for the [^14^C] work except only cold d-Gal was used. Data from three independent repeats are shown and the error bar uses sem.

The sequestered Und-PP-linked oligosaccharides were most likely trapped on the cytosolic face of the membrane for several reasons. Firstly, there is substantial genetic and biochemical evidence that the highly variable Wzx proteins function as the cognate repeat-unit flippase [27,29] and each acts only for an appropriate Und-PP-linked oligosaccharide or several variants [13,17, 21, 26, 30]. Secondly, ectopically overexpressing the *wzx* gene or switching to an appropriate Wzx can reverse the growth defect and repair the O-antigen biosynthesis pathway, including in group B1 used in this work [13,21, 26]. To validate this further, we rearranged the cell envelope with repeated sonication. Both the WT and the ∆*abe* strains were effectively lysed after sonication (Fig. 3a), with viable counts reduced >10^5^ (Fig. 3b). This method creates segments of the cytoplasmic membrane with materials from one leaflet of the membrane being adjacent to materials from the opposite leaflet with the same segment of membrane or between segments during the vesiculation process [31]. Such rearrangement would facilitate the release of sequestered oligosaccharides through WaaL ligase activity, leading to a significant decrease in [^14^C] d-Gal counts in the n-butanol extract.

Samples were subjected to repeated sonication, and changes in [^14^C] d-Gal counts in the purified LPS and n-butanol extractable fractions were assessed. The sonication resulted in a 43% reduction in [^14^C] d-Gal signals in the n-butanol extract from the ∆*abe* strain compared to the non-sonicated control (ratio paired t-test, *P*=0.0100) (Fig. 3c). In contrast, there was only a minor change in the [^14^C] d-Gal counts in the n-butanol extract from the WT strain after sonication (ratio paired t-test, *P*=0.4982) (Fig. 3d). These results strongly suggest that the sequestration of dead-end intermediates occurs specifically at the cytosolic face of the cell membrane.

## Discussion

The Und-P lipid carrier is crucial for the biosynthesis of peptidoglycan and surface polysaccharides in most bacterial species. The WecA-initiation pathway is a well-characterized example that is prevalent in *E. coli* (including *Shigella*) and *S. enterica*, where the Und-PP-linked *N*-acetylglucosamine (GlcNAc) product can be channelled into ECA or O-antigen production [32,33]. It has long been envisaged that the glycosyltransferases (GTs) would constitute a multi-protein complex. Under normal conditions, the pool of lipid carriers is likely regulated by the activity of specific GTs at the committed steps. In the GlcNAc-initiated pathway, the direction of synthesis is also complicated by the ratio of WecA recruited to respective pathways. The use of group B O:4 in this work evaded this complication to allow a direct assessment of Und-P sequestration. Defects in Und-P utilization typically result in poor growth, cell lysis and morphological abnormalities [10,16,18]. Previous studies have highlighted the role of Wzx flippase substrate preference in understanding Und-P sequestration and its detrimental effects on cells [13,14, 17, 20, 21, 26]. However, the direct assessment of Und-P sequestration has been challenging. The only direct evidence available is LC-MS data from methanol–chloroform–water extracted lipids in mutants of the ECA biosynthetic pathway [23]. Here, we utilized strains with defects in O-antigen repeat unit structure to capture Und-PP-linked O-antigen intermediates and assessed their sequestration.

Osborn and co-workers assayed the formation of Und-PP-linked O-antigen polymers using radioactively labelled nucleotide sugars with cells lysed via repeatedly sonication. In this case, the vesicles subsequently spontaneously generated between membrane fragments, bringing the initial GT and Wzy polymerase to the same leaflet for the Und-PP-linked oligosaccharide assembly and polymerization reactions to occur [34]. We leveraged the capacity of this approach that stalled Und-PP-linked intermediate could be alleviated by the exposure to WaaL ligase if the sequestration took place at the cytosolic face of the membrane. Interestingly, the conversion of the Und-PP-linked oligosaccharide into the LPS after sonication appeared insignificant in both the WT and ∆*abe* strains (Fig S1A, available in the online Supplementary Material). However, we anticipated that this would be the case, given the LPS fraction contains ~20-fold higher levels of radioactivity than the extracted Und-P pool in the ∆*abe* strain (Fig S1B). To this effect, any surplus [^14^C] signals in the LPS fraction derived from the alleviated Und-P would be deemed marginal. While our approach is limited to the accessible concentration and levels of [^14^C] d-Gal, we supplemented the quantitative assay with standard LPS gel migration analysis using samples prepared from cultures enriched with 25 mM d-Gal (~ 5 000 000-fold higher than the qualitative assay). Interestingly, compared to boiled samples for which all enzymatic activities were inactivated during the heat lysis treatment, those pretreated with sonication for which enzymatic activities remained active displayed marked enhancements in the levels of LPS containing short O-antigen repeat units, irrespective of the extraction procedures used (Fig S2).

The group B1 ∆*abe* strains showed tenfold higher counts for the n-butanol extracts relative to their respective parental strains. Kahne *et al.* reported a similar level of Und-P accumulation (16-fold) when lipid II translocation was blocked in a MurJ variant [35]. It should be noted that the kinetics of O-antigen precursor translocation and the subsequent incorporation into LPS are highly efficient in the WT. As such, Und-P pool turnover would occur at a faster pace during the preparation of the WT sample than in strains defective in translocation. Note also that the extracted Und-PP-linked O-antigen pool of the ∆*abe* strain, unlike the WT, would be primarily unpolymerized due to inaccessibility to Wzy polymerase. In such cases, the 10–16-fold sequestration of Und-PP-linked material required for cell death is likely an overestimate. Overall, these observations support that Und-P is available only in adequate (or slightly above demand) amounts to accommodate levels required for peptidoglycan and polysaccharide pathways. Recent evidence suggests a feedback control mechanism involving the MraY enzyme, which catalyses the production of lipid I, in that the enzyme possesses a potential lipid II-binding cavity on its extracellular face that is proposed to stall lipid II production in response to excess unincorporated peptidoglycan subunit [36]. Hence, regulated flow through shared pathways either by a feedback response as discussed with the MraY example or allocated competitively would be instrumental for bacterial survival, and these elements will need further investigation.

## supplementary material

10.1099/mic.0.001530 Uncited Fig. S1.

## Abbreviations

ECA: *Enterobacteriaceae* common antigens
GlcNAc: *N*-acetylglucosamine
GTs: glycosyltransferases
Und-P: undecaprenyl phosphate
