# Supplementary material for: Sequestration of dead-end undecaprenyl phosphate-linked oligosaccharide intermediate
Source: Microbiology (Reading). 2025 Jan 31;171(1):001530. doi: 10.1099/mic.0.001530 (PMC11784914; doi:10.1099/mic.0.001530)
Supplement: Uncited Fig. S1. [file mic-171-01530-s001.pdf]

SUPPLEMENTAL MATERIAL

Sequestration of dead-end undecaprenyl phosphate-linked oligosaccharide intermediate

Yaoqin Hong[1,2] and Peter R. Reeves[,2,*]

[1]Discipline of Biomedicine and Molecular Biology, College of Public Health, Medical and Veterinary Sciences, James Cook University, Queensland 4006, Australia
[2]School of Life and Environmental Sciences, The University of Sydney, New South Wales 4006, Australia

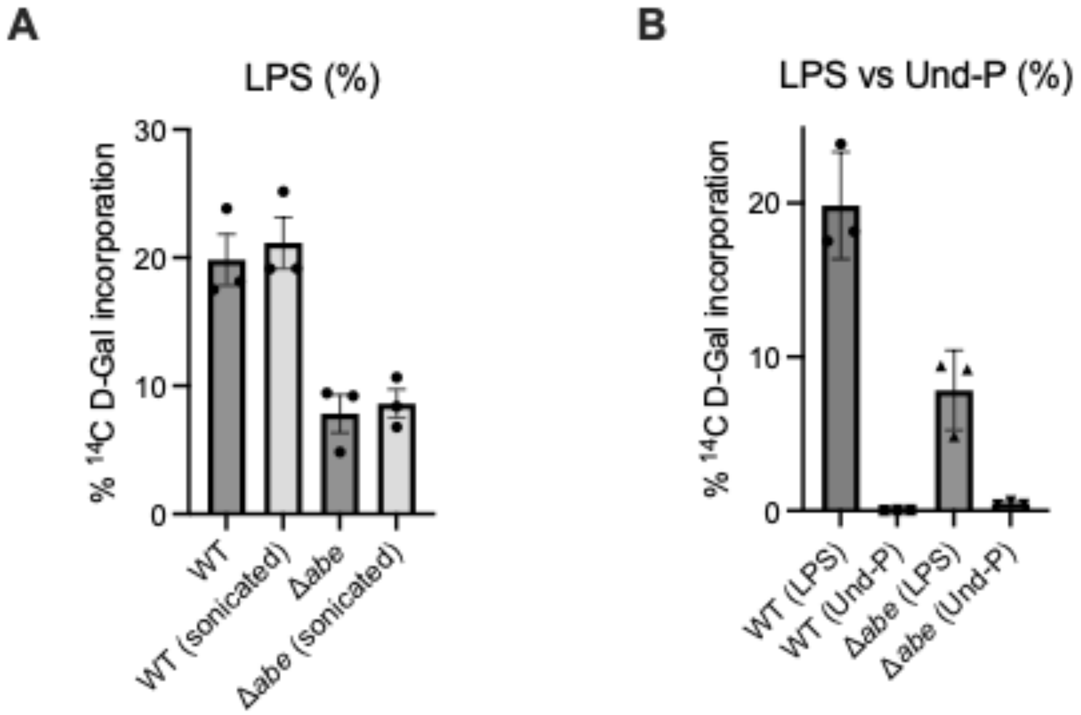

Fig S1. Effect of sonication-trigged membrane rearrangement on the [^{14}C] counts of LPS fraction. A, Lack of noticeable increment in extracted LPS counts after sonication; B, Relative [^{14}C] D-Gal uptake into LPS and Und-P fractions, respectively. Note that the unsonicated LPS and n-butanol extractable pool counts were the same datasets as Fig 2 and Fig 3 in the main text.

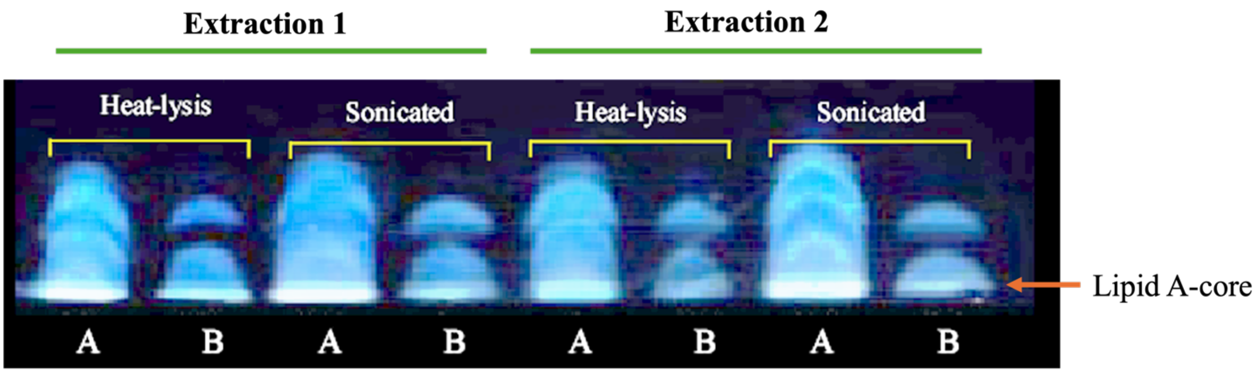

Fig S2. LPS profiles of *S. enterica* sv. Typhimurium Δ*galE* Δ*abe* mutant, strain P9529 after sonication or heat-lysis visualised on SDS-PAGE. The Δ*abe* strain was grown in depleted media until OD_{600} 0.45. D-Gal was then added to 25 mM for 30 min. The same batch was pelleted, resuspended in 80 mM Tris-acetate (pH 8.0), 10 mM MgCl2 and 1 mM EDTA. The samples were then divided into equal lots and lysed by either boiling or sonication. The lysed samples were then subjected to two independent extraction procedures, including phenol water (A) and phenol-chloroform (B) extractions. Equivalent sample amounts derived from two separate and independent samples (Extraction 1 & 2) were examined by silvered-stained SDS PAGE as described by Hong *et al.* (2012). Phenol water extraction was performed as described previously by Hong *et al.* (2012) and phenol-chloroform extraction was described by Guard-Petter *et al.* (1995). Note that the image was reversed to improve clarity.

REFERENCES

Guard-Petter, J., Lakshmi, B., Carlson, R., and Ingram, K. (1995) Characterization of lipopolysaccharide heterogeneity in Salmonella enteritidis by an improved gel electrophoresis method. *Appl Environ Microbiol* **61**: 2845-2851.

Hong, Y., Cunneen, M.M., and Reeves, P.R. (2012) The Wzx translocases for Salmonella enterica O-antigen processing have unexpected serotype specificity. *Molecular Microbiology* **84**: 620-630.
